# One Step in Advance

**Published:** 1892-03

**Authors:** Oliver Martin

**Affiliations:** Ottawa, Ont.


ARTICLE III.

### ONE STEP IN ADVANCE.

BY OLIVER MARTIN, L. D. S., OTTAWA, ONT.

Although much has been said on the subject of filling teeth, it is one that is always of deep interest to the dentist, every point made is noted, and a trial given, to test the merits of the new idea, whether theory or actual work. After so many years of experience, it would appear almost impossible to add anything new of importance in the method of filling teeth, for, after all, it is but a method of preserving teeth as long as possible, yet it has taxed the ingenuity of the ingenious dentist from its conception to the present time. He is not yet satisfied, it is not likely he ever will be.

This feature in the dentist's character is commendable. It stimulates him to greater perfection, and still greater as he understands that there is no such thing as absolute perfection; everything must go on and improve for ever.

In accordance with these remarks, I wish to advance one step more in the manner of filling teeth, as I have no fear of the dentists becoming wearied of the subject.

The use of gold foil appears to prove itself superior for filling teeth, but it is difficult of manipulation, as the least moisture spoils its cohesive properties. To overcome this, the patient's mouth is filled with napkins, paper, rubber dams, propped open with corks, jack-screws, until the eyes bulge out as if in the last agonies,—and the dentist, in a nervous state at the rapid flow of saliva, knowing full well that after all his work of preparation he is likely to lose a good filling before he can finish it, as it will take an hour. It is to overcome these difficulties in large gold fillings, at the same time remove a tax from the patients, that will bring more than many thanks for the dentist.

I now speak of large gold fillings. Foil will always be used in small fillings of ten or fifteen minutes, but when it comes to a half hour, the system I shall propose is preferable.

We will take, for example, a patient: three large gold fillings are to be inserted; it will take at least three sittings, but often more. Prepare the cavities only at the first sitting, take an impression of them (wax is as good as the other materials), place your temporary filling of wax or gutta-percha, and dismiss your patient without fatigue to the patient or to the dentist of consequence. The difference is, you intend to make a perfect filling for these three teeth in your laboratory at your leisure, independent of saliva. Mix enough plaster with fine plumbago to hold the plumbago together, and set; oil the wax impression as for an ordinary plaster cast. While you are preparing the gold, place your mould to dry on the oil stove; use 22, fine gold, which is coin. It is fine enough for any filling; it will never change, but it will resist more attrition. The mould can be made of the plaster and plumbago alone, but small flasks in the form of the ordinary moulding would be better. The plaster mould should be tied together with a narrow band of tin, or stove-pipe iron. Your three moulds ready, and placed in convenient position, melt your gold in a small crucible and pour quickly; it will not do as well to have two fillings in one mould, as one or the other is likely to be imperfect. If the mould is well made, the filling requires but little trimming; the sprue cut off, it is ready. As every dentist understands moulding, it is unnecessary to explain it. You now have three fillings ready. When the patient makes her appearance, you feel no dread of saliva; the first filling is freed from the tooth, the cavity cleansed of wax particles, you try the fit of your gold filling, and trim so it will enter the cavity readily. Test the articulation and trim accordingly. Everything being ready, a single napkin is all that will be needed. Mix your oxide chloride of zinc, or other of the plastic fillings—the oxide-chloride adheres to metals very firmly, and is preferred on that account. Where it does not cause too much irritation, line the cavity with it, using but little, but enough to be pressed out. When you press in your solid gold filling, there will remain just enough to fill the imperfect adaptation of the gold, and this is what is most needed. All the fine pits and lines of the cavity are filled, which is seldom accomplished with foil, up to the thin edge of the enamel. Owing to the nice fit of the gold, there is but little oxide exposed around the walls of the cavity to be acted upon by secretions, and the action is not the same, owing to the presence of gold, which plays with zinc. It will endure as much as the tooth substance, unless too much is exposed owing to a bad fit; in such a case, remove a little of the oxide, and fill with foil, which is the work of but a few moments. In finishing the filling do so from the centre of the filling towards the surrounding walls, as it spreads the gold over the thin line of the oxide. The antagonizing of the teeth will also spread the gold. Still there will be little danger of decomposition, as it is but a film, and preserves bone very well.

Whatever defect occurs to a filling of this kind is from the exterior. In the manner your three gold fillings are inserted at one sitting, with but little trouble to your patient, and the satisfaction to the dentist that he has inserted solid gold filling that will stand the test of time, with but one napkin. In tha case of incisors, bicuspids, or any of the front teeth where the cavity is arc-shaped and cutting edge is required, it will be safer to drill a small hole through that part of the filling where, when it comes in contact with the cavity of the tooth, a shallow hole can be drilled into the tooth that will not endanger it, or give the patient much pain, of two or three threads; than tap the end of a gold wire the size of the hole in the filling, and screw it in gently. After the oxide has hardened, cut it off and finish. Such a filling will stay, and stand to cut in molars. Where it would be deemed necessary, two screws can be inserted. In this way a large gold filling can be inserted in a useful third molar of the lower jaw. In many cases amalgam is objected to on account of the mercury, but tin is as favorable in the mouth as gold; they can be cast in the same manner, with little trouble, as it melts easily. The technical points are hardly necessary to mention, as all practitioners are acquainted with them. But the principle of forming the fillings for large cavities in the laboratory instead of in the patient's mouth, doing away with the dangerous hand pressure, the terrible mallet, according to the patient's version of it, the fatigue of opened mouth for an hour and a half and more, clamps, dams, cords, weights, all to fight away the saliva in order to produce a solid gold filling; this is the step in advance. As we improve we can simplify. There are many advantages in this step in advance filling,—it is a solid gold, or tin, filling with more power of resistance than what can be produced with foil. It can be held in any form of cavity, with wire screws if it comes out; it can be replaced with fresh cement, at little expense to the patient. This will be quite an inducement to patients to have their teeth filled with gold. The foil filling will not allow to be drilled into; this will, and the many devices that can be brought into play on account of this privilege will soon manifest itself to the dentist, who will feel confident of securing his fillings. The cavity of the tooth should be shaped so as to allow the impression to come out without drag on the wax. Time will be saved by this in the adjustment of the filling. Now we can place a solid gold filling in any tooth, in any part of the mouth, with less trouble, and a more permanent operation, than a small foil filling in the front teeth.—*Dominion Dent. Journal.*

